# Oil composition determines microbial preference for carbon source between hydrocarbons and Necromass in water droplets within crude shale oil

**DOI:** 10.1093/ismeco/ycag242

**Published:** 2026-08-24

**Authors:** Qiao-Hui Wang, Yi-Fan Liu, Biao Wang, Qin Xiao, Lei Zhou, Shi-Zhong Yang, Ji-Dong Gu, Bo-Zhong Mu

**Affiliations:** State Key Laboratory of Bioreactor Engineering and School of Chemistry and Molecular Engineering, East China University of Science and Technology, Shanghai 200237, P.R. China; MOE-Engineering Research Center of Microbial Enhanced Oil Recovery, East China University of Science and Technology, 130 Meilong Road, Shanghai 200237, P.R. China; State Key Laboratory of Bioreactor Engineering and School of Chemistry and Molecular Engineering, East China University of Science and Technology, Shanghai 200237, P.R. China; MOE-Engineering Research Center of Microbial Enhanced Oil Recovery, East China University of Science and Technology, 130 Meilong Road, Shanghai 200237, P.R. China; State Key Laboratory of Bioreactor Engineering and School of Chemistry and Molecular Engineering, East China University of Science and Technology, Shanghai 200237, P.R. China; Research Institute of Petroleum Engineering Technology, Jiangsu Oilfield, Sinopec, Yangzhou 225009, P.R. China; MOE-Engineering Research Center of Microbial Enhanced Oil Recovery, East China University of Science and Technology, 130 Meilong Road, Shanghai 200237, P.R. China; State Key Laboratory of Bioreactor Engineering and School of Chemistry and Molecular Engineering, East China University of Science and Technology, Shanghai 200237, P.R. China; MOE-Engineering Research Center of Microbial Enhanced Oil Recovery, East China University of Science and Technology, 130 Meilong Road, Shanghai 200237, P.R. China; State Key Laboratory of Bioreactor Engineering and School of Chemistry and Molecular Engineering, East China University of Science and Technology, Shanghai 200237, P.R. China; MOE-Engineering Research Center of Microbial Enhanced Oil Recovery, East China University of Science and Technology, 130 Meilong Road, Shanghai 200237, P.R. China; State Key Laboratory of Bioreactor Engineering and School of Chemistry and Molecular Engineering, East China University of Science and Technology, Shanghai 200237, P.R. China; MOE-Engineering Research Center of Microbial Enhanced Oil Recovery, East China University of Science and Technology, 130 Meilong Road, Shanghai 200237, P.R. China; Department of Environmental Science and Engineering, Guangdong Technion—Israel Institute of Technology, 241 Daxue Road, Shantou, Guangdong 515063, P.R. China; State Key Laboratory of Bioreactor Engineering and School of Chemistry and Molecular Engineering, East China University of Science and Technology, Shanghai 200237, P.R. China; MOE-Engineering Research Center of Microbial Enhanced Oil Recovery, East China University of Science and Technology, 130 Meilong Road, Shanghai 200237, P.R. China

**Keywords:** oil reservoir, shale oil, water droplet, microbial community, metabolism

## Abstract

Water droplets entrapped in crude oil have recently been recognized as unexpected but metabolically active microbial habitats in shale oil reservoirs. However, the key environmental drivers that shape the community composition and metabolic profiles of these droplet microorganisms remain poorly understood. Here, we investigated three shale oil samples from Jiangsu oilfield, all of which contained abundant water droplets (>10^5^ droplets/ml oil) of ancient formation water origin. Integrated microscopy, metagenomics, and metabolomics revealed that the physicochemical properties of the enclosing oil, primarily API gravity and viscosity, are associated with droplet size and may influence the droplet microbiomes. These factors appear to jointly drive a fundamental metabolic dichotomy, where in light oil with low-salinity water, communities are enriched with hydrocarbon degraders together with molecular signatures of active petroleum hydrocarbon metabolism. In more viscous, light-to-medium oil with high-salinity water, however, communities shift towards necromass recycling and strong genetic adaptations to osmotic stress. Our findings demonstrate that microbial life in water droplets enclosed in shale oil is widely found in the shale oil reservoirs examined, and selected by carbon quality and environmental pressure. This study provides a mechanistic framework for understanding microbial ecology and biogeochemical processes in shale oil reservoirs and offers insights for microbial enhanced oil recovery in heavy oil reservoirs.

## Graphical Abstract

**Graphical Abstract:**
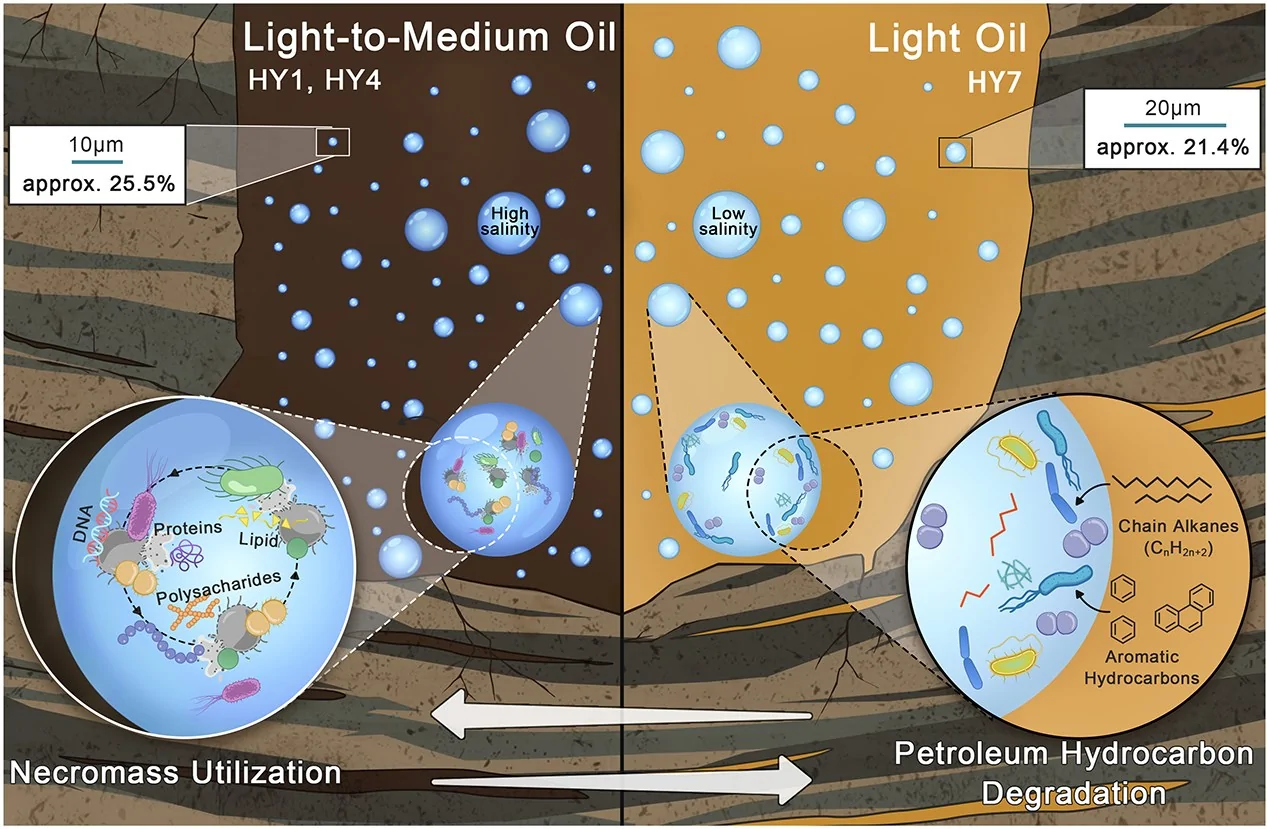

## Introduction

Oil reservoirs represent one of the most extensive and energy-rich deep subsurface ecosystems on the earth, harboring abundant and diverse microbial communities [1–3]. The microorganisms in these extreme environments are an important component of the deep biosphere and play a crucial role in biogeochemical cycles [4]. Understanding the ecology of these microbial communities is therefore necessary for geomicrobiology by providing fundamental insights into subsurface life, biogeochemical processes, and potential biotechnological applications such as enhanced oil recovery. Conventionally, microbial life in subsurface oil reservoirs primarily inhabits the aqueous phase (water leg) [5]. Hence, investigations of microorganisms in oil reservoirs mainly used production water and formation water as a proxy for the water leg. Analyses of these aqueous samples have consistently shown that the microbial community composition is shaped by multiple geochemical factors (salinity, sulfate, and nitrate concentrations, etc.) and physical reservoir conditions (temperature, permeability) [6, 7]. For instance, high salinity of the water leg exerted a strong selective pressure, enriching halotolerant taxa such as *Halanaerobiales* [8]. Similarly, sulfate-rich conditions typically promoted the dominance of sulfate-reducing bacteria like *Desulfovibrio* [9]. However, the traditional view of oil and water as separated phases has overlooked the physicochemical properties of oil itself, particularly the differences between heavy and light crude oils, as direct selective forces on microbial communities residing in the water leg.

However, the water leg is not the only habitat for microorganisms in oil reservoirs. Recent investigations have further reported that microbial life is present in minuscule water droplets trapped in oil samples from the shale oil reservoir and asphalt lakes, and those water droplets have an ancient origin [10, 11]. Metagenomic and metabolomic analysis revealed a high diversity of microbial communities in the water droplets, which exhibited notable metabolic activity [10, 11]. These communities generally included heterotrophic and anaerobic taxa such as *Rhodobacteraceae, Sphingomonadales, Methanomicrobiales*, and *Bacteroidales* [12]. Multi-omics evidence showed that these microorganisms can utilize dissolved hydrocarbons and necromass-derived organic compounds for growth, supporting several metabolic pathways such as fermentation, syntrophy, and methanogenesis. The abundant water droplets and microorganisms within them significantly increase the potential for oil biodegradation in shale oil reservoirs. These findings provide a crucial information for understanding microbial adaptation strategies in minuscule oil-entrapped water droplets.

Critical gaps remain in understanding the ecological principles governing these unique microhabitats. It is unclear whether the observed metabolic strategies based on primary carbon and energy sources, hydrocarbon degradation and necromass recycling, are universally conserved or environmentally selected in response to specific physicochemical gradients within the oil system [10, 12]. Specifically, the relative importance and interactive effects of key gradients, such as oil composition and droplet salinity, in shaping the taxonomic composition and functional assembly of droplet microbiomes have not been systematically deciphered. Crude oil composition is especially critical, as it governs hydrocarbon bioavailability and droplet physics, two of the most important factors that exert strong selective pressure on droplet microbiomes. A lack of this mechanistic understanding limits our ability to predict microbial activity, ecological dynamics, and their biogeochemical roles across diverse oil reservoirs.

To address these gaps, this study employed an integrated multi-omics approach to analyze three shale oil samples from the Jiangsu Oilfield, which represented a natural gradient in oil composition and water salinity. We aimed to elucidate how the interplay between oil properties and water chemistry determines the size distribution of entrapped droplets, selects for distinct microbial taxa, and ultimately drives the partitioning of core community metabolic strategies between hydrocarbon utilization and necromass recycling.

## Materials and methods

### Oil and water sampling

Shale oil samples were obtained from three oil wells in the same block (HY1, HY4, and HY7) in Jiangsu oilfield, which cannot be explored by water-flooding (Table S1) [13]. Oil samples were collected and transferred immediately into sterile 500 ml glass bottles that had been pre-flushed with pure nitrogen. Each bottle was filled completely and sealed with butyl rubber stoppers to prevent oxygen intrusion. The fracturing fluid samples were also handled in an identical manner, representing the chemical mixture as injected into the formation. All samples were transported to the laboratory after collection and stored at 4°C, subsequent processing and analysis were completed within 2 days.

To investigate different aqueous compartments, we obtained four types of water samples from the shale oil: (i) Gravitationally separated water (GSW) was collected from the shale oil by natural gravity during transport and storage; (ii) Individual water droplets (IWDs) were isolated according to a method previously established: spread the shale oil on aluminum foil, and individual water droplets (0.1–1 μL) were sampled with a 2.5-μL pipette [11]; (iii) Separated water (SW) was obtained by incubating shale oil in a water bath at 46°C for 24 h to facilitate droplet coalescence and sedimentation, followed by collection of the bottom water phase, representing aggregated water droplets; and (iv) To assess water-soluble compounds present in the oil phase, dehydrated shale oil was vigorously mixed with sterile water (1:1, *v/v*), shaken for 5 min at 25°C, and the aqueous phase was collected after phase separation as extracted water (EW).

### American petroleum institute gravity of shale oil samples

The American petroleum institute (API) gravity of the shale oil samples was determined according to ASTM D5002. Oil samples were first heated to 40°C in a thermostatically controlled chamber to ensure the complete dissolution of wax crystals and achieve a low viscosity. Density measurements were performed using a digital density analyzer (Anton Paar DMA 4501), each sample was analyzed in triplicate.

### Viscosity of shale oil samples

The viscosity of raw and dehydrated shale oil samples was measured using a rotational rheometer (HAAKE MARS III, Thermo Fisher Scientific, Germany) equipped with MARS Temperature Module Controller. A titanium rotor (P35 Ti L) with a measuring gap of 0.300 mm was employed. The measurement program consisted of two steps: (i) an equilibration stage at 20 ± 1°C for 5 min under constant stress (τ = 0 Pa); and (ii) a continuous temperature scan from 20°C to 120°C at a constant shear rate of 10 s^−1^ over 10 min.

### GC–MS analysis

0.5 ml of dehydrated oil was mixed with 3 ml of n-hexane, vortexed and centrifuged at 10 000 rpm for 15 min. The solvent phases of HY7 were directly filtered through 0.22-μm-pore-size membrane filter before injection, whereas the solvent phases of HY1 and HY4 were diluted 1:20 before filtration and injection. GC–MS analysis was conducted on an Agilent 5975C GC-MSD system (Agilent Technologies, USA). Samples were injected using an automated GC sampler with a 1 μL injection volume in split mode (split ratio 30:1). An HP-5 capillary column (30 m × 0.32 mm × 0.17 μm) was used with He as the carrier gas. The inlet temperature was set to 320°C, and the oven temperature program was held at 120°C for 3 min, ramped at 10°C/min to 300°C and held for 20 min. The MS transfer line was maintained at 280°C, and the quadrupole was set to 150°C. Mass spectra were acquired in full-scan mode (*m/z* 24–550).

### Water content in oil samples

The water contents of the three shale oil samples were all determined in triplicate using Karl–Fischer Titration (831 KF Coulometer, Metrohm, Switzerland).

### Water droplets distribution in the shale oil

The spatial distribution of water droplets in the shale oil was visualized via X-ray computed tomography (CT) scanning (nanoVoxel-2000, Sanying, China) at a scan voltage of 90 kV. Shale oil was filled directly into 2 ml Eppendorf tubes for analysis, 3D images were taken with a spatial resolution of 5.18 μm.

### Stable isotope composition of water samples

The ^18^O and ^2^D isotopic compositions of SW, GSW and fracturing fluid were measured using a GLA431-TLWIA liquid water isotope analyzer (ABB LGR, Canada) with a precision of 0.1‰ for δ^18^O and 0.4‰ for δ^2^D. All analyses were performed at least in triplicate and results were expressed as the deviation in permille (‰) between the sample and Standard Mean Ocean Water using the delta notation. Further details of the database can be found in Supplementary Text.

### Ion composition of water samples

The SW, GSW, and fracturing fluid were also analyzed in triplicate with ion chromatography (ICS-5000+, Dionex, Canada). Anion separation was performed using an AS11-HC 4 mm column with a potassium hydroxide eluent gradient consisting of 0.8 mM and 20 mM KOH, delivered at a flow rate of 1.0 ml/min. For cation analysis, separation was achieved on an IonPac CS12A column (4 × 250 mm) with an isocratic elution of 18 mM methanesulfonic acid, also maintained at a flow rate of 1.0 ml/min.

### Cell counting in individual water droplets

First, 1 μL of water droplets was diluted with sterile water to a final volume of 10 μL. Subsequently, cells within the sample were stained by adding 1 μL of a 1% crystal violet solution. Cell counting was performed using a cell counting chamber (177-112C, Watson, Japan) under a phase contrast microscope (CX43, Olympus, Japan). A negative control, consisting of sterile water processed in parallel under identical conditions, was included in each experiment.

### Cell membrane integrity in water samples

Shale oil samples were spread on glass cell culture dishes (801001, Nest, China) to expose the water droplets enclosed in shale oil. Cells in the water droplets were stained by adding 0.2 μL stain reagents (LIVE/DEAD BacLight bacterial viability kit, ThermoFisher, USA), both stain reagents, Syto 9 and propidium iodide (PI), were diluted 20 times [14]. Incubated it in the dark for 5 min at room temperature. The cell membrane integrity was assessed using confocal laser scanning microscopy (CLSM). The microscope (A1R, Nikon, Japan) equipped with 100× Apo TIRF NA 1.49 oil objective, 488-nm and 561-nm argon lasers (see Supplementary Text for more details).

### ATP quantification

Cell membranes in water droplets were broken by adding 5 μL pooled water droplets to 25 μL UltraLyse 7 reagent (Deposit & Surface Analysis Test Kit DSA-25, LuminUltra, Canada). The ATP concentration was measured in triplicates by EQP-PBM-PAC luminometer (LuminUltra, Canada).

### DNA extraction

SW and IWDs were collected for DNA extraction. Specifically, IWDs were collected for 16S rRNA gene amplicon sequencing. SW was collected as a proxy for the pooled water droplets to obtain sufficient biomass for metagenomic analysis, with three SW samples serving as biological replicates for each reservoir. For the HY7 reservoir, the extracted DNA from one of the SW samples did not meet the required quality requirements for library preparation, and consequently, this reservoir yielded only two successful metagenomic replicates. Genomic DNA extraction was performed with AxyPrep™ Bacterial Genomic DNA Maxiprep Kit (Axygen Biosciences, United States). DNA concentrations were determined using a Qubit 2.0 Fluorometer (BioRad, Hercules, United States) and confirmed with agarose gel electrophoresis (0.8%, w/v). All DNA samples were stored at −20°C for later sequencing.

### 16S rRNA gene amplicon sequencing analysis

DNA sequencing service was provided by the Center for Applied Genomics at the Hospital for Sick Children (Toronto, ON, Canada), using an Illumina NovaSeq X Plus platform. DNA samples were amplified with PCR primers 515F/806R for bacteria and PCR primers Arch519F/Arch915R for archaea. Amplicon sequences processing was performed by QIIME2 [15]. After FASTQ files were imported, the barcode and primers were trimmed, and the amplicon paired-end reads were merged and quality-filtered using TrimGalore. Amplicon sequence variants (ASVs) inferred using fastp (v0.23.4) and the DADA2 pipeline in R (v1.30.0). Taxonomic assignment of the final ASV set was performed against the SILVA v138 reference database.

### Metagenomic sequencing and analysis

For metagenomic analysis, libraries were constructed using the Illumina Nextera XT DNA Library Prep Kit and sequenced to generate 150 bp paired-end reads. The raw data of metagenomic sequencing were quality-filtered and adapters were removed using TrimGalore v0.6.5 (default parameters). Quality-filtered reads were co-assembled using metaSPAdes (v3.9.0) [16]. MAGs generated by MaxBin2 (v2.2.6) [17], MetaBAT2 (v2.12.1) [18], and CONCOCT [19] were pooled into DAS-tool for dereplication [20]. The completeness and contamination of MAGs were estimated using CheckM (v1.1.2), and only MAGs with >50% completeness and <10% contamination were retained for further analysis. Taxonomic assignments of the MAGs were performed with GTDB-Tk (v1.3.0) and verified by using the 16S rRNA gene sequences aligned on SILVA r138 [21]. For quantitative comparisons of functional gene abundances between samples, a complementary read-based profiling approach was employed. Clean reads were mapped to this gene set using Salmon (v1.10.3) in mapping-based mode. The representativeness of the assembled scaffolds was assessed by mapping reads back to scaffolds using BBMap (v39.81), yielding recruitment rates of 92.3%–99.6% (see Supplementary Text for more details).

### Microbial community diversity analysis

Microbial diversity and community similarity analyses were conducted in R using the vegan package (v2.7-1). *α-*diversity was quantified using the Shannon, Simpson, and richness indices. For each group, the mean, standard deviation, median, and range of these indices were calculated. Differences in *α-*diversity among groups were evaluated using one-way analysis of variance (ANOVA). *β-*diversity was assessed using Bray–Curtis dissimilarities computed with vegdist. Principal coordinates analysis (PCoA) was performed for ordination. Differences in community composition across groups were tested using permutational multivariate analysis of variance (PERMANOVA) implemented in adonis2 (999 permutations).

### Redundancy analysis and Mantel test

Redundancy analysis (RDA) was performed to examine the relationships between microbial community composition and environmental variables using the vegan package (v2.7-1) in R. A constrained ordination model was constructed with the RDA function, and sample scores, species scores, and environmental vectors were extracted for visualization. Species–environment associations were interpreted based on the magnitude and direction of species vectors along environmental gradients.

To further quantify the relationships between environment-associated ASVs identified in RDA and environmental factors, Mantel tests and correlation analyses were conducted in R. Pearson correlations among environmental variables were calculated using the linkET package (v0.1.0), while Mantel correlations between the ASV abundance distance matrix and environmental distance matrix were computed using the mantel function in vegan (v2.7-1).

### ESI FT-ICR MS analysis

Components in dehydrated oil, EW and water droplet samples were detected via a 7.0 T Bruker SolariX FT-ICR mass spectrometer with negative-ion mode ESI. The water samples were centrifuged at 12 000 rpm for 20 min at 4°C, and the supernatant was collected and diluted in methanol, while dehydrated oil was dissolved in toluene and diluted with methanol. Mass spectra were acquired at *m/z* 100–1200 with co-added 200 continuous scans to improve the signal-to-noise ratio. Instrument settings are provided in the Supplementary Text.

### UHPLC–MS/MS analysis

For metabolite profiling, a Vanquish UHPLC system coupled to a Q Exactive Plus Orbitrap mass spectrometer (Thermo Fisher Scientific, Germany) was used [22, 23]. Following concentration by centrifugation, 10 μL water droplets were vacuum-dried and reconstituted in 50 μL of methanol. After centrifugation (12 000 rpm, 4°C, 20 min), 5 μL supernatant was subjected to chromatographic separation on an ACQUITY UPLC BEH C18 column (100 × 2.1 mm, 1.7 μm, Waters) maintained at 45°C, with a flow rate of 0.4 ml/min. Mass spectrometric detection employed a heated electrospray ionization source in negative-ion mode with a spray voltage of −2.8 kV and a capillary temperature of 320°C (Data acquisition see Supplementary Text).

## Results

### Physicochemical properties of shale oil samples

The quality of the three shale oil samples was characterized based on their API gravity. HY1 and HY4 shale oil samples were classified as light-to-medium oil (LMO) with API gravities of 32.90 and 32.07, whereas the HY7 shale oil was light oil (LO) with an API gravity of up to 46.36. The viscosity of HY7shale oil was 232.6 mpa·s as measured at 40°C, while HY1 and HY4 oil samples exhibited higher viscosities of 1188 mpa·s and 1192 mpa·s, respectively (Fig. S1).

**Figure 1:**
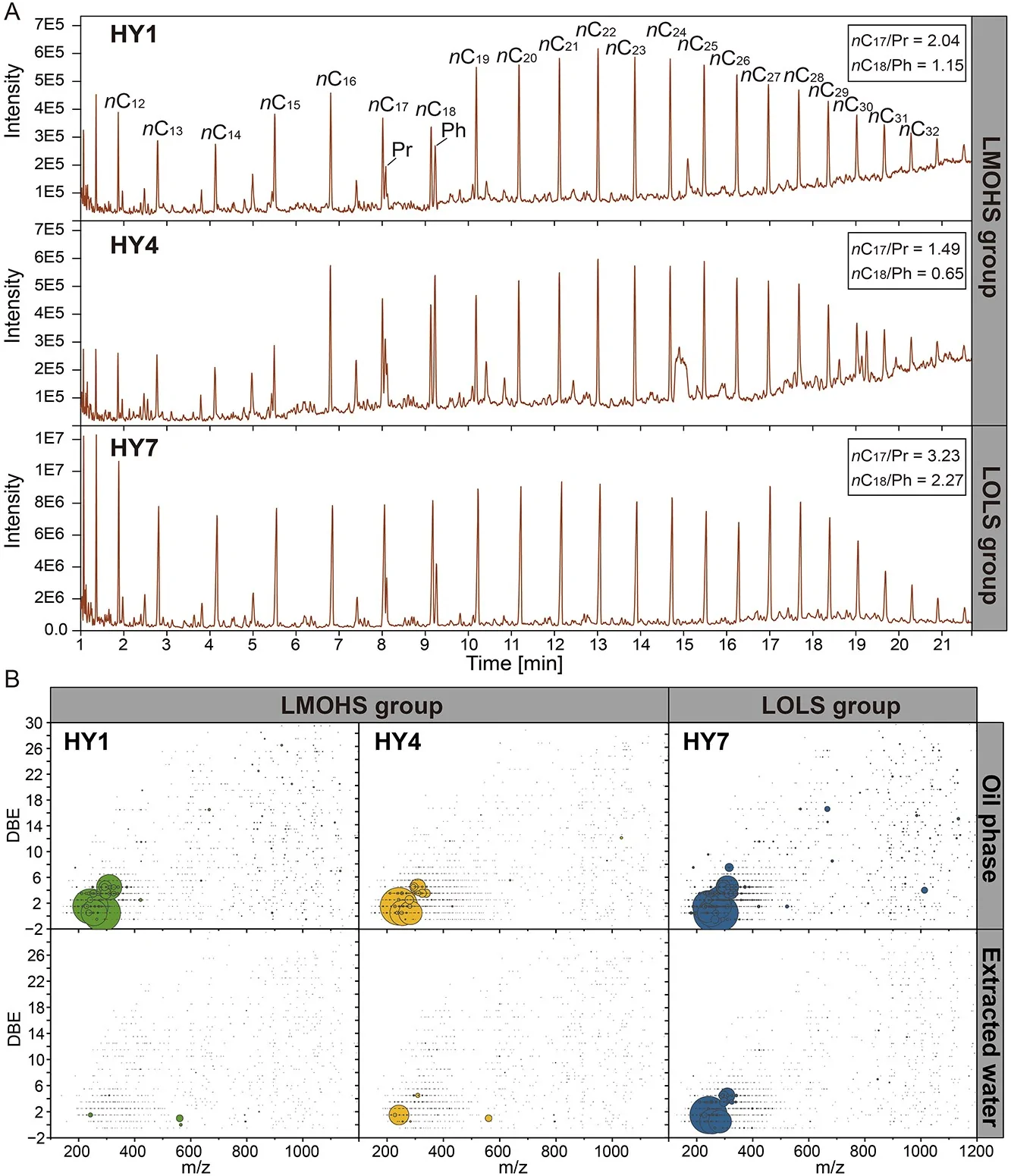
Physicochemical properties of shale oil samples. (A) Gas chromatogram of shale oil samples dissolved in *n*-hexane. Pristane and phytane are marked in the spectrum, the ratios of *n*C_17_/pristane (*n*C_17_/Pr) and *n*C_18_/phytane (*n*C_18_/Ph) are shown in the upper right corner. (B) Distribution of molecular formulas identified from the extracted water and the oil samples, plotted as double bond equivalents (DBE) versus mass. Each point represents an assigned molecular formula detected by ESI FT-ICR MS, circle areas indicate relative abundance.

GC–MS analysis of the three shale oil samples showed distinct hydrocarbon profiles (Fig. 1A). For HY7 shale oil, intense peaks in the early retention window (<10 min) revealed abundant light *n*-alkanes (C*_n_, n* < 19). In contrast, HY1 and HY4 shale oils displayed markedly weaker light-hydrocarbon signals, along with a rising baseline at later retention times, suggesting a predominance of heavy components [24]. Specifically, the lower *n*C_17_/pristane (*n*C_17_/Pr) and *n*C_18_/phytane (*n*C_18_/Ph) ratios in HY1 and HY4 shale oil indicated a greater extent of biodegradation in these samples [25].

ESI FT-ICR MS analysis was applied to reveal molecular characteristics of the dehydrated oil, and the EW samples were also analyzed to evaluate water-soluble compounds in the oil phase (Fig. 1B). All oil samples were predominantly composed of low-unsaturation, low-molecular-weight compounds (Table S2, DBE < 6 and *m/z* < 400). Among these, compounds in the HY7 shale oil exhibited the lowest average carbon number of 23.8. Notably, the EW from HY7 contained more compounds with low unsaturation and low molecular weight compared to HY1 and HY4, suggesting that the HY7 shale oil contained more light water-soluble components (Fig. 1B).

Based on their physicochemical properties, the crude oil samples HY1 and HY4 demonstrated notable similarities, whereas HY7 exhibited distinct characteristics. Accordingly, utilizing the API gravity classification, we categorized samples from HY1 and HY4 as LMO group and those from HY7 as the LO group.

### Distribution and source of water droplets enclosed in shale oil

**Figure 2:**
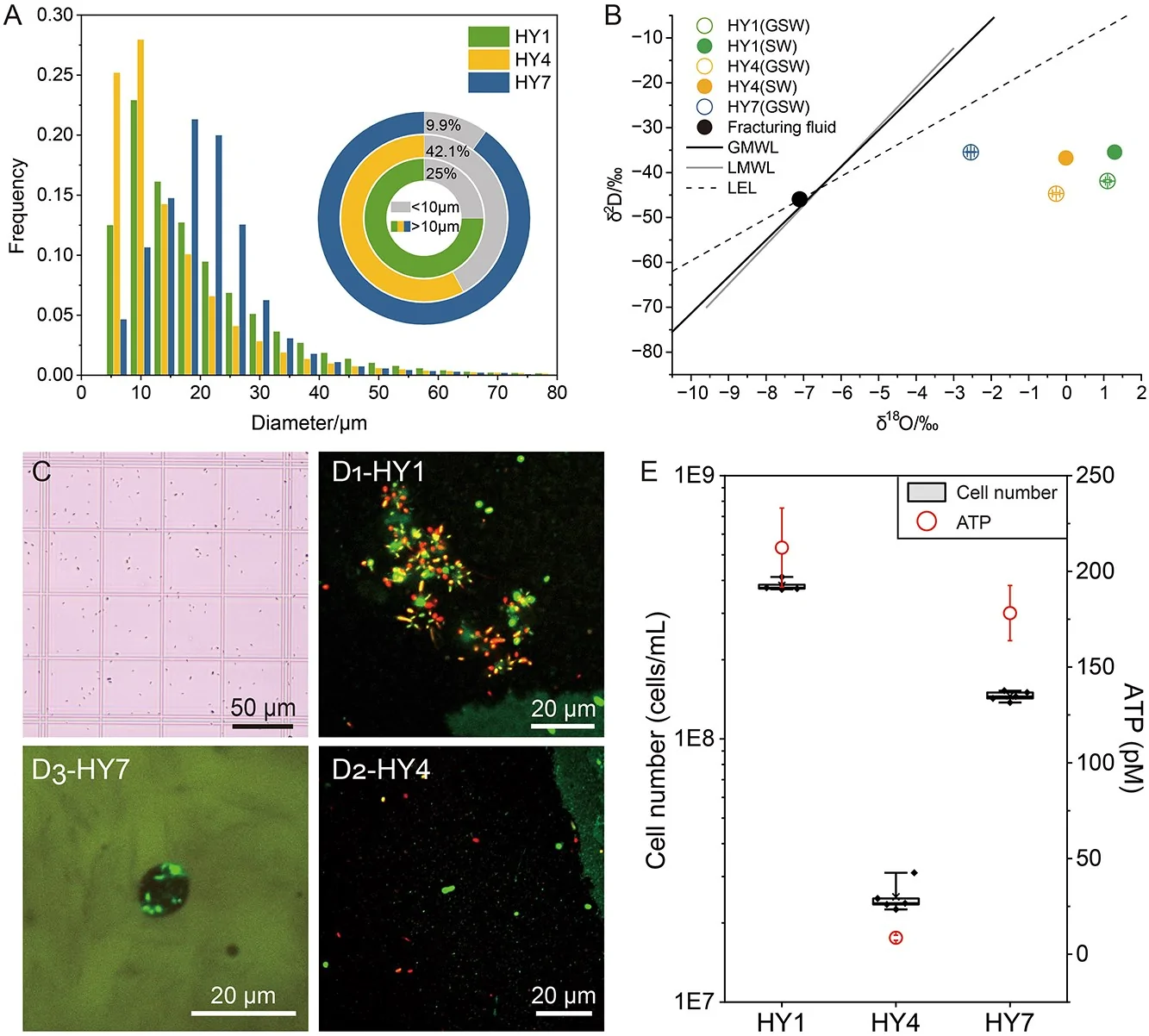
Size distribution and stable isotope composition of water droplets, the number and viability of microorganisms inside the water droplets. (A) Size distribution of water droplets obtained by CT scanning. The ratio of the number of water droplets with diameters greater or less than 10 μm to the total number of droplets is shown in the insert. (B) *δ*^18^O and *δ*^2^D of separated water (SW, solid particle), gravitationally separated water (GSW, hollow particle) and fracturing fluid (black solid circle) compared to the global meteoric water line (GMWL, black solid line), the local meteoric water line (LMWL, gray solid line), and the local evaporation line (LEL, black dotted line). (C) Phase-contrast microscope image of cells in the water droplets from HY1. (D) CLSM image of cells within water droplets enclosed in three shale oil samples, green fluorescent signals represent live cells, red fluorescent signals represent dead cells. (E) Box plots of total cell counts and measured ATP contents of isolated water droplets from the three different shale oil samples.

These two groups also exhibited different water contents and droplet distribution. Water content exceeded 7.0% in the LMO group but was only 2.3% on average in the LO group (Table S3). CT imaging confirmed the presence of water droplets in all samples, with concentrations ranging from 0.23 × 10^6^ to 4.04 × 10^6^ droplets/ml oil (Figs 2A and S2, Table S4). In the LMO group, most droplets were around 10 μm in diameter, whereas in the LO oil, the diameter of most droplets was predominantly larger, centered around 20 μm (Fig. S2; Kruskal–Wallis test, *P* < 0.001).

The origin of water entrapped in shale oil was determined by analyzing water stable isotopes and ion concentrations [10]. For the HY7 sample, however, the volume of SW was insufficient for isotope analysis, so only its GSW was included in the isotopic dataset. The *δ*^2^D and *δ*^18^O of all the SW and GSW samples clearly deviated from the global meteoric water line and local meteoric water line, indicating a different origin from meteoric water (Fig. 2B). In contrast, the fracturing fluid samples plotted close to both the global and local meteoric water lines. This finding further supports that the water in shale oil reservoirs has not been contaminated by fracturing fluids during exploitation. Additionally, the *δ*^18^O in water samples shifted to the right compared to the local evaporation line, suggesting that these water samples cannot originate from the evaporation of local surface water [26]. Both SW and GSW from the same oil reservoir also showed a consistent composition and similar concentrations of major ions, aligning with their shared origin (Table S5). Compared with the fracturing fluid, all the SW and GSW samples exhibited high salinity with Na^+^ and Cl^−^ as the dominant ions. Combined with the results of stable isotope and ion composition analyses, it can be inferred that water droplets in shale oil samples primarily originated from ancient formation water [10, 11].

**Figure 3:**
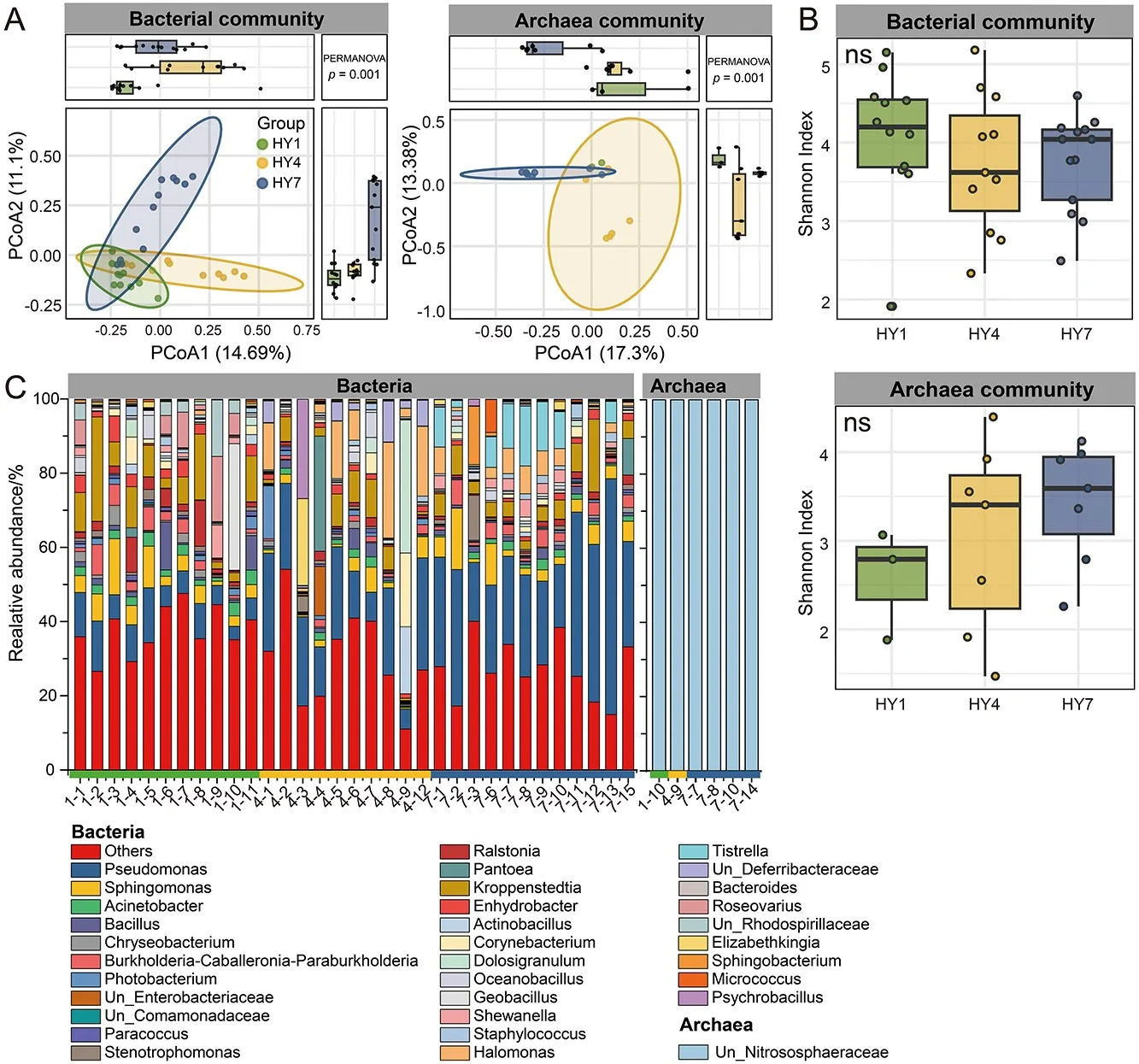
Microbial diversity and microbial community structure in water droplets from HY1, HY4 and HY7. (A) PCoA plot based on Bray–Curtis distances showing the *β*-diversity differences among water samples, with statistical significance evaluated by PERMANOVA. ns: not significant, i.e. *P* > 0.05, ^*^: *P* < 0.05, ^**^: *P* < 0.01, ^***^: *P* < 0.001. The boxplots on the top and right show the distribution of samples along each PCoA axis for each group. (B) *α*-diversity of microbial communities in water samples from different oil reservoirs, represented by the Shannon index. (C) Microbial communities in water droplets at the genus level.

Furthermore, the water chemistry differed notably between the LMO and LO groups. The LMO group exhibited markedly greater salinity, and water samples from low-salinity HY7 showed elevated CO_3_^2−^ levels and abundant organic acids, especially in the SW sample, which may reflect more severe hydrocarbon biodegradation in the HY7 oils [27]. Therefore, based on the characteristics of both the oil and water samples, samples from HY1 and HY4 were further classified as Light-to-Medium oil with High-Salinity water (LMOHS) group, while those from HY7 were classified as Light oil with Low-Salinity water (LOLS) group. This classification provides a clear framework for subsequent analysis of microbiome differences.

### Microbial distribution and community composition in water droplets

Water droplets harbored abundant and morphologically diverse microbial communities. Hemocytometer quantification revealed total cell counts of (0.25–3.83) × 10^8^ cells/ml in all water droplets (Fig. 2C&E), with HY7 displaying the highest cell counts and HY1 showing the lowest cell concentration. No significant difference in cell numbers was observed between the LMOHS group and the LOLS group. Remarkably, these values are about 1000 times higher than the cell concentrations (10^4^–10^5^ cells/ml) commonly found in other production water samples [5, 28–30].

CLSM imaging confirmed the prevalence of live over dead cells in the water droplets, with viable cells in LOLS group predominantly located at the oil–water interface (Fig. 2D). This interfacial location for microorganisms increases their hydrocarbon accessibility [31–34], which was consistent with previous findings [10, 14]. However, live cells were found closer to dead cells and located in the center of water droplets in the LMOHS group. Additionally, ATP in water droplets from all samples was detected with concentrations approximated to 10^−22^ mol ATP per cell (Fig. 2E). Collectively, these findings demonstrate that water droplets within the shale oil harbored dense and diverse microbial communities, characterized by exceptionally high cell density and viability.

**Figure 4:**
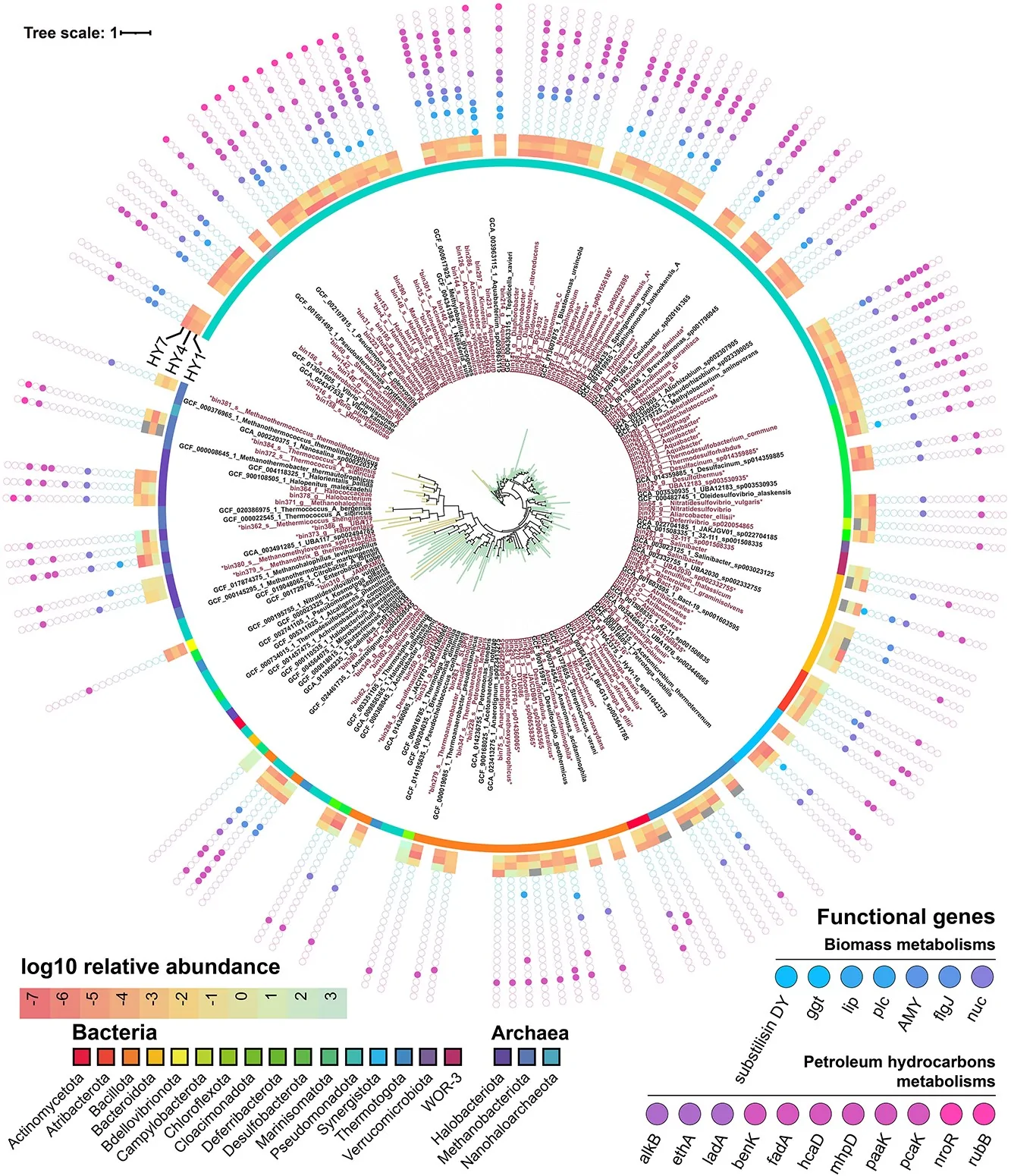
Phylogenetic analysis and MAG-based functional annotation of microbial communities in water droplets. Maximum-likelihood phylogenetic tree **(**built by IQ-TREE) of 117 MAGs (red text), asterisks indicate HQ MAGs. The different colors in the innermost circle illustrate genus-level annotation. The relative abundance of each MAG is shown in the middle circle. Annotated functional genes involved in biomass metabolisms and petroleum hydrocarbon metabolisms were marked as dots on the outermost ring. MAG-based gene annotations shown here are intended for taxonomic association of functions.

As revealed by 16S rRNA gene survey, the microbial community composition in water droplets differed significantly between the two groups (Fig. 3A). The microbial communities (bacteria and archaea) in LMOHS water droplets clustered more closely together, whereas they were significantly separate from those in LOLS group in the PCoA plot (PERMANOVA: *P* = 0.001). However, analysis of alpha diversity revealed no substantial differences in Shannon index between the two groups (Fig. 3B and Table S6). Other alpha diversity indices like Simpson and Richness indices also showed the same result (Fig. S3), indicating that the significant compositional difference likely resulted from species replacement rather than a loss or gain of overall diversity. The dominant bacterial members in water droplets were *Pseudomonas* and *Kroppenstedtia*, which were commonly detected across all samples from both groups (Fig. 3C). In contrast, *Halomonas* and *Roseovarius* were predominantly detected in LMOHS water droplets, while *Tistrella* was highly abundant only within the LOLS group. An intra-group difference was found in the LMOHS group, for instance, members of the *Deferribacteraceae* were present exclusively in the droplets from HY4. Notably, members from these genus/families have also been found in other oil reservoir samples or petroleum-contaminated soil samples [10, 35–39]. Metagenomic sequencing supported the 16S rRNA gene survey results, identifying the same dominant bacterial genera, including *Halomonas, Desulfonauticus*, and *Pseudomonas* (Fig. 4). Archaea were only detected in 6 out of 39 water droplets, suggesting a low abundance of archaea in these microhabitats. This result is consistent with the previous shale oil sample [10]. Compared with the bacterial community, the archaeal community was highly simplified, being composed solely of the family *Nitrososphaeraceae* in water droplets. However, metagenomic sequencing revealed a broader range of archaeal taxa, including Halobacteriota and methanogenic lineages (Table S7).

**Figure 5:**
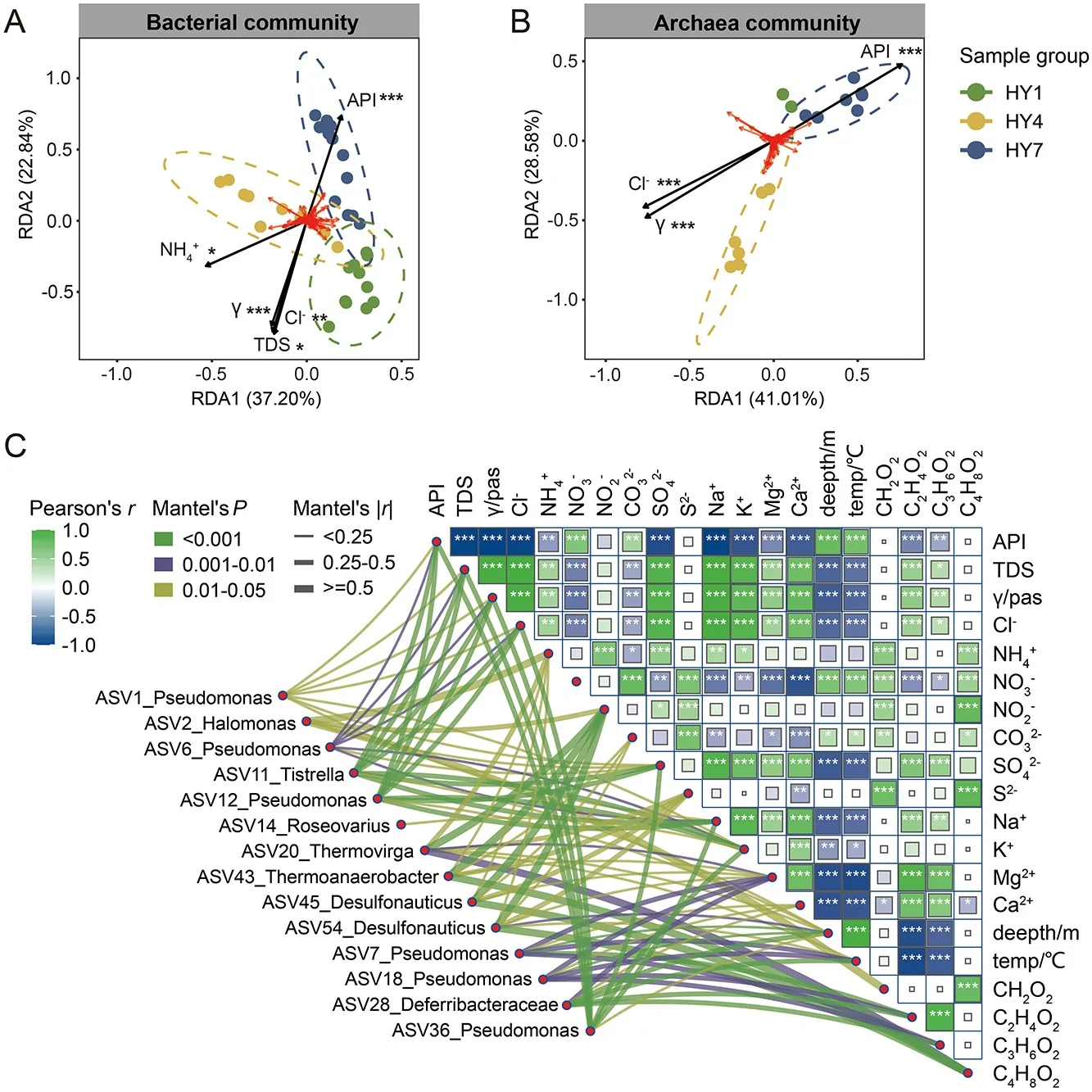
Correlation analysis between the microbial community structure and environmental factors in water droplets from HY1, HY4 and HY7. (A, B) Distance-based redundancy analysis (db-RDA) of the microbial community with environmental factors. ANOVA-like permutation test was performed for RDA to assess the significance of the factors, ^*^: *P* < 0.05, ^**^: *P* < 0.01, ^***^: *P* < 0.001. (C) The heatmap displays pairwise Pearson correlations among environmental factors. Network diagram shows the results of Mantel tests assessing the correlation between each environmental factor and the bacterial community dissimilarity (Bray–Curtis distance based on ASVs). Edge width corresponds to Mantel’s |*r*| statistic for the corresponding distance correlations, edge color denotes the statistical significance.

The distance-based redundancy analysis (db-RDA) showed that the discrimination of the microbial communities in the two groups is mainly driven by API gravity and viscosity (*γ, P* < 0.001), and followed by Cl^−^, NH_4_^+^ and TDS (*P* < 0.05) (Fig. 5A and B and Table S8). To further examine the relationships between key environmental factors and specific microbial taxa, we selected ASVs that exhibited significant contributions to the db-RDA ordination and differential abundance between the two groups for Mantel test analysis (Fig. 5C). The result showed that the relative abundances of *Pseudomonas* and *Tistrella* were negatively associated with API gravity and viscosity (Mantel test: *P* = 0.001).

### Potential microbial metabolisms in water droplets

Metagenomic analysis of the SW samples generated a total of 117 MAGs with completeness ranging from 50% to 100% and contamination ranging from 0% to 9.65%, representing phyla of Pseudomonadota, Halobacteriota, Desulfobacterota, and Bacillota (Fig. 4 and Table S7). Among these, 73 MAGs met the high-quality (HQ) criterion (completeness >90%, contamination <5%), accounting for over 62% of all recovered MAGs. We identified several extracellular hydrolases and transporters for proteins/peptides, polysaccharides, lipids/fatty acids, and nucleotides, and intracellular degradation pathways for the corresponding monomer in multiple MAGs (Figs 4 and S4). This result aligns with the previous hypothesis that microorganisms in oil-embedded water droplets may obtain carbon and energy sources by recycling detrital biomass [40]. Previous studies have also shown that the microbial communities in such water droplets degrade surrounding petroleum hydrocarbons [10–12, 14]. Accordingly, genes involved in both anaerobic and aerobic degradation of petroleum hydrocarbons were detected in 37 HQ MAGs (representing 30 genera, Table S9), including putative *assA* (alkylsuccinate synthase, Table S10), *bssA* (benzylsuccinate synthase), *alkB* (alkane 1-monooxygenase), *ladA* (long-chain alkane monooxygenase), *catA* (catechol 1,2-dioxygenase), and *benA* (benzoate 1,2-dioxygenase subunit alpha), demonstrating a substantial potential for petroleum hydrocarbon degradation encoded within diverse members [41].

**Figure 6:**
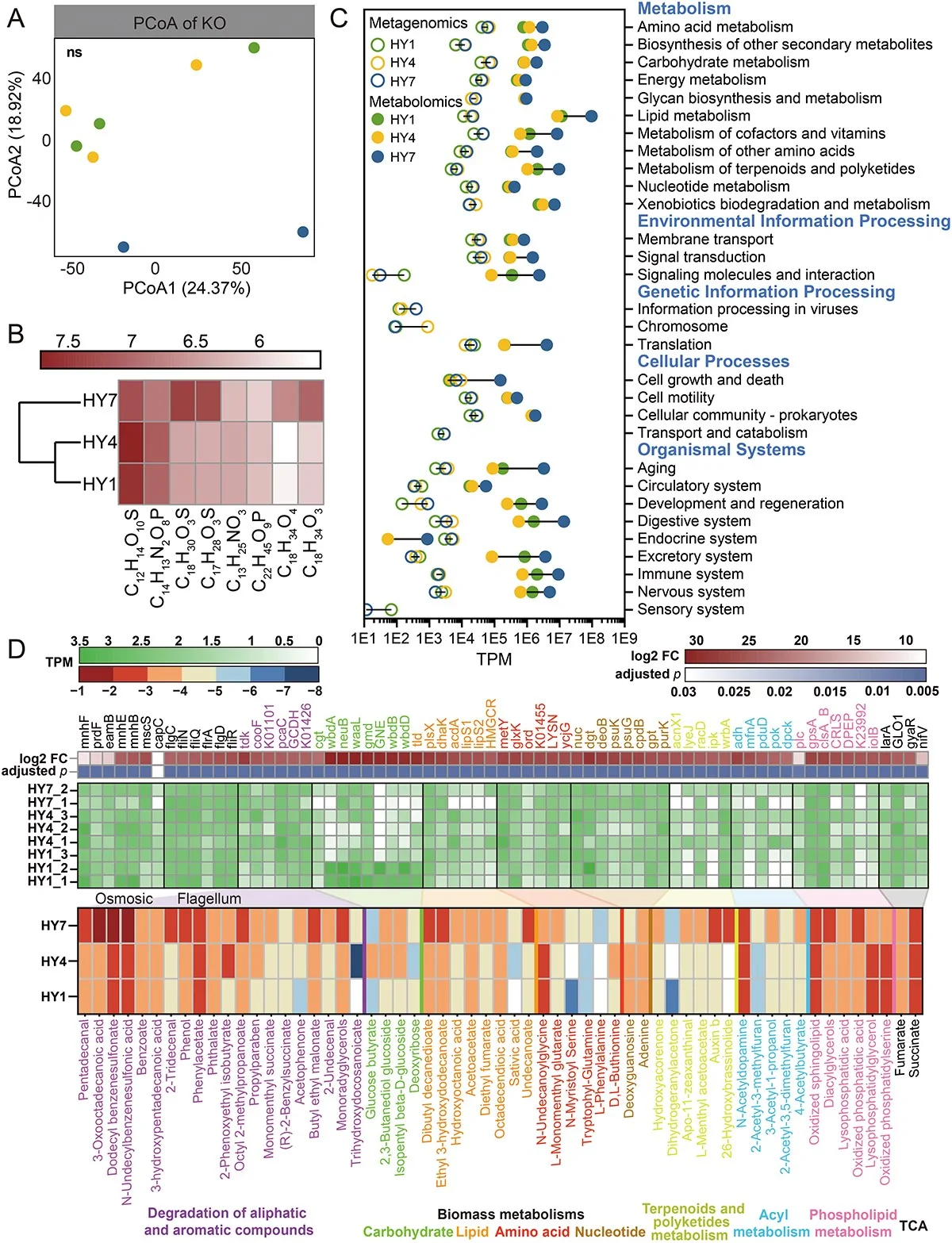
Integrative analysis of metagenomic (read-based functional profiles) and metabolomic datasets. (A) PCoA plot based on KEGG ortholog (KO) in water droplets from HY1 (green), HY4 (yellow) and HY7 (blue) shale oil samples, significance was assessed by PERMANOVA. (B) Clustered heatmap of metabolites detected in water droplets from three shale oil samples. (C) Metabolic pathway enrichment analysis based on metagenomic and metabolomic datasets. (D) Heatmap showing the relative abundances of key functional genes and metabolites across samples, associated metabolic pathways are color-coded and annotated below. Differential abundance of functional genes between groups was analyzed using DESeq2 (v1.42.0) with the Wald test [64]; Log2FC of genes in different samples and adjusted *P*-values are reported.

To compare functional gene abundances quantitatively between groups, we performed read-based functional profiling against the non-redundant gene set. KEGG functional annotation revealed that the dominant pathways across all samples were related to amino acid metabolism, carbohydrate metabolism, cofactor, and vitamin metabolism and nucleotide metabolism (Fig. 6C). Furthermore, PCoA analysis revealed a functional differentiation between the two groups, wherein the LMOHS samples were similar to each other but distinct from the LOLS sample (Fig. 6A) [42]. Genetic profiling revealed a significant enrichment in the LOLS group of key genes (all adjusted *P* < 0.05) associated with alkane hydrocarbon degradation (*alkB*, Log2FC = 5.4). However, the genes encoding monooxygenase specific for long-chain alkanes (*ladA*) were found in low abundance and evenly distributed in both groups (adjusted *P* > 0.05). This is reasonable since both groups contain heavy hydrocarbons, including long-chain alkanes. The LOLS group also harbored higher abundances of genes related to fatty acid transport (*fadL*, Log2FC = 24.85) and degradation (*fadN*, Log2FC = 12.62), and fumarate reduction (*frdB*, Log2FC = 13.97) (a complete list of genes’ full names could be found in Table S11). In contrast, genes involved in carbohydrate metabolism (*cgt*, Log2FC = 21.97), nucleotide metabolism (*nuc*, Log2FC = 25.88) and phospholipid metabolism (*plc*, Log2FC = 10.82) were more abundant in water in LMOHS group (all adjusted *P* < 0.05). Moreover, aligning with the high salinity in LMOHS water, genes associated with osmotic stress response (*mnhF*, Log2FC = 9.92; *capC*, Log2FC = 7.36, all adjusted *P* < 0.05) were also enriched in LMOHS group. Notably, some genes displayed very large Log2FC values, reflecting near-absence of the gene in one group compared to detectable abundance in the other. Such values should therefore be interpreted as semi-quantitative indicators of presence/absence patterns rather than precise fold-change estimates.

**Figure 7:**
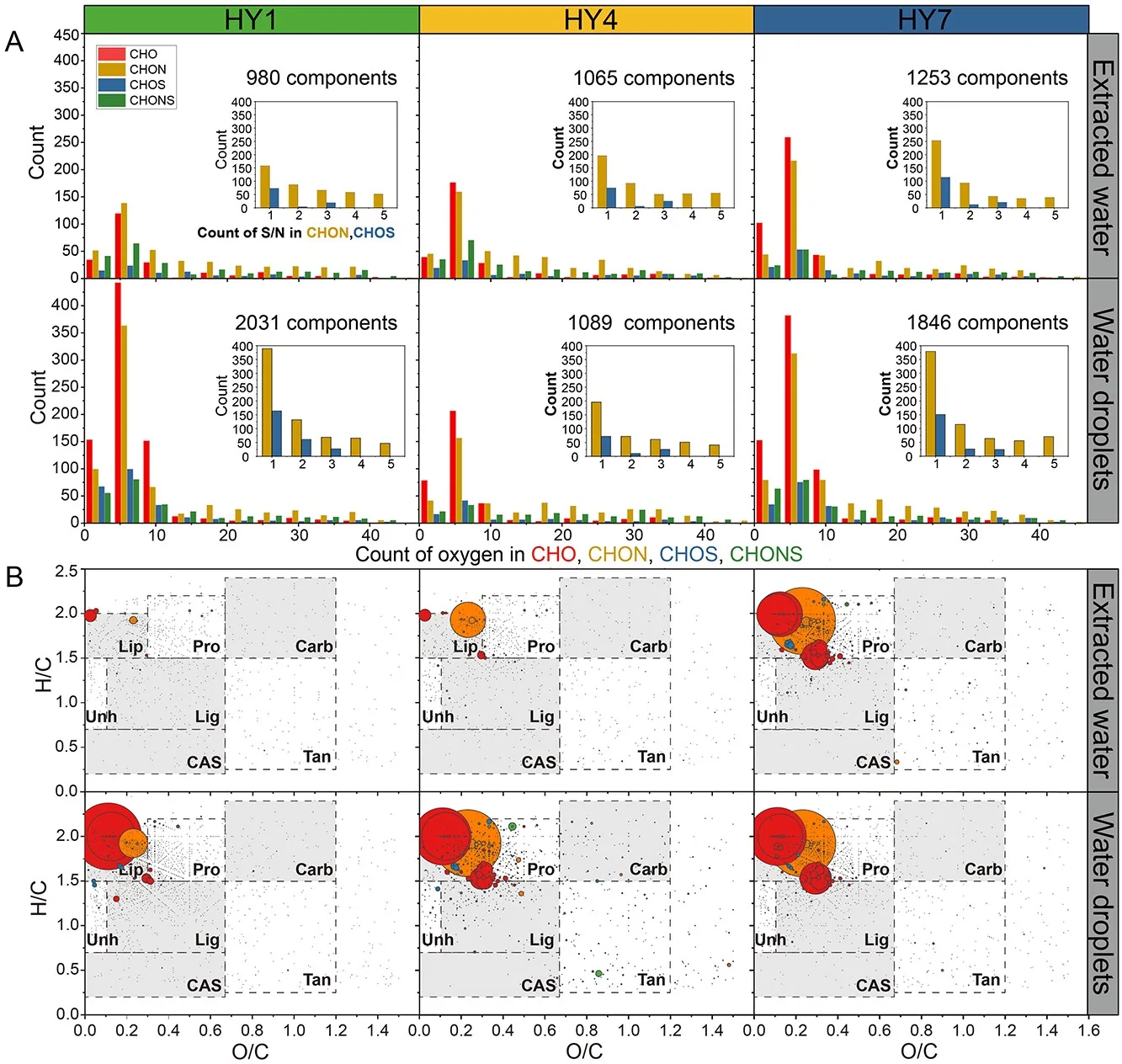
MS-derived molecular compositions of water droplets and extracted water samples from HY1 (left panel), HY4 (middle panel) and HY7 (right panel), circle areas indicate relative abundance. All formulas are color-coded according to CHO (red), CHON (yellow), CHOS (blue) and CHONS (green) molecular series. (A) Count and distribution of molecular series with different numbers of oxygen atoms in water samples, the embedded section shows the ratio of O/N and O/S in CHON, CHONS and CHOS compounds, respectively. (B) van Krevelen diagrams with boxes overlain on the plots show major biomolecular compound classes, including Lipid-like (Lip), Protein-like (Pro), Carbohydrate-like (Carb), Unsaturated hydrocarbon-like (Unh), Lignin-like (Lig), Tannin-like (Tan), Condensed aromatic structures-like (Cas).

To verify the proposed microbial metabolic process of microorganisms within water droplets, the molecular compositions of water droplets and EW samples were identified by ESI FT-ICR MS. The identified molecular formulas were classified into four major categories based on their elemental composition: CHO-only, CHON-only, CHOS-only, and CHONS (Fig. 7). Water droplet samples displayed greater molecular formula richness, while EW samples consistently yielded fewer detectable formulas (Table S2). Specifically, oxygen-rich compounds (O_4_–O_7_ classes) showed higher relative abundance in water droplets compared to EW, largely corresponding to different carboxylic acids (Fig. 7A) [43, 44]. Saturated fatty acids (C_18_H_36_O_2_ and C_16_H_32_O_2_) dominated in water droplet samples, which are common components of microbial membranes and can also be produced by petroleum hydrocarbon degradation [45]. Notably, in the LMOHS group, lipid-like CHO compounds were more abundant in water droplets than in EW, probably due to necromass release (Fig. 7B).

The molecular structure of metabolites in water droplets can be determined through the UPLC-MS/MS analysis. Notably, a greater number of compounds were detected in water droplets from the LOLS group than in those from the LMOHS group (Table S12). Hierarchical clustering of all detected metabolites revealed that metabolites in LOLS water droplets were similar to but different from the LMOHS group (Fig. 6B). Potential intermediates involved in petroleum hydrocarbon degradation showed a higher relative abundance in LOLS water droplets (Fig. 6D, 15.2%) compared to those from the LMOHS group (1.3% on average). This pattern was corroborated by the enrichment of specific aromatic hydrocarbon biodegradation intermediates in HY7 water droplets, including benzoate, phenol and (R)-2-benzylsuccinate [23, 46]. Conversely, potential intermediates related to biomass metabolism showed an opposite trend, with higher relative abundances in the LMO group (2.7% on average) [23, 46]. This apparent shift toward biomass utilization was further supported by the elevated levels of phospholipid metabolism intermediates in the LMO group, such as oxidized phosphatidic acid and diacylglycerols, implying a microbial strategy of necromass recycling via cellular membrane degradation.

## Discussion

In this study, we demonstrate how microscale water droplets in shale oil reservoirs serve as unique, self-contained microbial habitats, thereby shaping microbial ecology and metabolic pathways. Each droplet harbors abundant and metabolically active microorganisms with densities exceeding 10^7^ cells/ml. Our integrated multi-omics approach reveals that the physicochemical properties of the surrounding oil phase and the encapsulated water itself profoundly shape not only the size distribution of these droplets but, more importantly, the microbial ecology and dominant metabolic pathways within them.

### The viscosity-dependent size distribution of entrapped water droplets in shale oil

The high number of water droplets entrapped in shale oil (>10^5^ droplets/ml oil), with an average diameter of 20 μm (Table S4), underscores their potential as significant microbial microhabitats. A key finding is that the viscosity of the shale oil exerts a primary control on droplet size distribution [47]. We propose that the lower viscosity of LO facilitated the coalescence of dispersed aqueous phases over geological time, leading to the statistically larger droplets observed compared to those in LMO [48, 49]. This viscosity-mediated control on droplet size has direct ecological implications: larger droplets in LO may present a greater oil–water interfacial area, potentially facilitating access to hydrophobic hydrocarbons for interfacial microorganisms [11].

Critically, these droplets are not extrinsic contaminants but relic formation waters. Their distinct isotopic signatures (*δ*^18^O enrichment) and ion compositions (high salinity), which clearly deviate from modern meteoric water and fracturing fluids (Fig. 2B), attest to an indigenous origin shaped by prolonged water–rock interactions under reservoir conditions [50]. Therefore, these droplets represent ancient, isolated aqueous micro-habitats that have co-evolved with the enclosing oil phase. This unique setting provides a stable interface for hydrocarbon–microbe interactions.

### Oil composition also dictates microbial carbon source preference

Our integrated metagenomic and metabolomic data reveal that the physicochemical properties of shale oil, primarily mediated by API gravity/viscosity, enforce a fundamental divergence in the metabolic strategies of the enclosed droplet microbiomes. This divergence manifests as a partitioning of primary carbon sources between exogenous petroleum hydrocarbons and endogenous necromass.

In the LOLS group, the higher bioavailability of light hydrocarbons appears to promote the enrichment of microorganisms at the oil–water interface and sustain a community strategy centered on hydrocarbon degradation [51]. The enrichment of genera like *Pseudomonas* and *Tistrella*, well known for their capabilities of hydrocarbon degradation and biosurfactant production (Fig. 5C) [52, 53], together with the annotation of genes encoding pathways for alkane/aromatic degradation (e.g. *badH, benA*) and biosurfactant production (*bgsAB*) within above two genera [53, 54], aligns with the concurrent accumulation of hydrocarbon degradation intermediates and biosurfactants (N-acyl amino acids) in the droplets (Fig. 6D) [55]. Collectively, these findings provided strong evidence for active hydrocarbon degradation in the water droplets. This activity could initiate a positive feedback loop, where biosurfactant production enhances emulsification, potentially further reducing local oil viscosity and sustaining a favorable environment for degraders [56].

Conversely, in the heavier and more viscous LMOHS oil, the depletion of light hydrocarbons and the reduced diffusivity imposed by higher viscosity have likely selected for a strategy of intense necromass recycling at the center of water droplets [57]. This adaptation is supported by the enrichment of putative degraders (e.g. *Thermovirga*) (Fig. S4), elevated functional genes involved in lipid, nucleotide, and peptide metabolism (e.g. *nuc*) (Fig. 6D), and a metabolome dominated by biomolecular intermediates such as fatty acids and amino acid derivatives [58]. Together, these lines of evidence point to a microbial community adept at scavenging and remineralizing detrital biomass. This strategy represents a remarkable adaptation to extreme nutrient limitation, efficiently retaining and cycling carbon and nutrients within the confined droplet ecosystem over geological timescales [40]. Moreover, the observed enrichment of heavy fractions and depletion of light alkanes in the oil reservoir likely resulted from prior biodegradation [5], suggesting that the reliance on necromass as a primary carbon source may reflect an evolutionary shift in substrate preference as the community adapted to dwindling hydrocarbon availability. It should also be noted that the current study only examined light and LMOs. Future work should intentionally include medium and heavy crude oil samples to test whether the substrate-partitioning patterns observed here are ubiquitous across a broader spectrum of oil qualities.

### Salinity acts as a key environmental filter selecting for halotolerant taxa and driving osmoadaptation

Clear differences in taxonomy were observed across the salinity gradient. LMOHS droplets were enriched with halophilic/halotolerant genera like *Roseovarius* and *Halomonas* [59, 60], while the LOLS community showed no comparable enrichment. Metagenomic analysis further uncovered the genetic basis for this divergence. Microbes in high-salinity water droplets harbored higher abundances of genes involved in osmotic stress adaptation, including *mnhF, mscS*, and *dhal* [61]. The enrichment of these genes indicated that high salinity exerts a strong selective pressure, favoring taxa equipped with diverse osmoprotection mechanisms [62, 63]. In contrast, the absence of dominance halophilic microorganisms and the lower abundance of osmotic stress-related genes in low-salinity water implied a milder environment, reducing the need for complex salt-adaptation strategies [60].

This study reveals that shale oil reservoirs harbor abundant indigenous water droplets that serve as naturally encapsulated microbial microreactors. Such microbial ecosystems are not randomly assembled but are predictably shaped by a hierarchy of oil and environmental characteristics. Oil physicochemical properties control droplet physical structure and hydrocarbon bioavailability, whereas water salinity acts as a primary filter selecting for osmotolerant taxa. The interplay of these factors drives a fundamental metabolic dichotomy, where hydrocarbon degradation prevails in LO with low-salinity water, while necromass recycling dominates in more viscous, LMO with high-salinity water. These coordinated adaptive responses, supported by consistent metagenomic and metabolomic evidence, highlight a sophisticated level of microbial ecological organization in deep-subsurface extreme environments. Beyond shale oil, our findings offer a conceptual framework for understanding microbial life in any water-in-oil emulsion system within the deep biosphere.

## Supplementary Material

Supplementary_material_ycag242

## Data Availability

The 16S rRNA gene amplicon sequences and metagenome short reads are available in the National Center for Biotechnology Information under BioProject PRJNA1073723 as BioSamples SAMN53472449**-**SAMN53472451.

## Supplementary material

Additional experimental details and parameters; physiochemical properties of analyzed samples; plots and datasets from metagenomic analysis; mass spectrometry results and visualizations (PDF). Supplementary material is available at *ISME Communications* online.
