# Supplementary material for: Oil composition determines microbial preference for carbon source between hydrocarbons and Necromass in water droplets within crude shale oil
Source: ISME Commun. 2026 Aug 24;6(1):ycag242. doi: 10.1093/ismeco/ycag242 (PMC13573599; doi:10.1093/ismeco/ycag242)
Supplement: Supplementary file 1 [file supplementary_material_ycag242.zip › SI_Wang_et_al_clean_ycag242.docx]

Supplementary information

**Oil Composition Determines Microbial Preference for Carbon Source Between Hydrocarbons and Necromass in Water Droplets Within Crude Shale Oil**

Qiao-Hui Wang^1,4^, Yi-Fan Liu^1,4,*^, Biao Wang^1,2,4^, Qin Xiao^1,4^, Lei Zhou^1,4^, Shi-Zhong Yang^1,4^, Ji-Dong Gu^3^, Bo-Zhong Mu^1,4,*^

^1^ State Key Laboratory of Bioreactor Engineering and School of Chemistry and Molecular Engineering, East China University of Science and Technology, Shanghai 200237, P. R. China

^2^ Research Institute of Petroleum Engineering Technology, Jiangsu Oilfield, Sinopec, Yangzhou 225009, P. R. China

^3^ Environmental Engineering Program, Guangdong Technion - Israel Institute of Technology, 241 Daxue Road, Shantou, Guangdong 515063, P.R. China

^4^ MOE-Engineering Research Center of Microbial Enhanced Oil Recovery, East China University of Science and Technology, 130 Meilong Road, Shanghai 200237, P. R. China

* Corresponding authors at:

East China University of Science and Technology, Shanghai 200237, China.

*E-mail address*: [liuyifan@ecust.edu.cn](mailto:liuyifan@ecust.edu.cn) (Yi-Fan Liu)

East China University of Science and Technology, Shanghai 200237, China.

*E-mail address*: [bzmu@ecust.edu.cn](mailto:bzmu@ecust.edu.cn) (Bo-Zhong Mu)

This PDF file includes:

Supplementary methods

Figures: S1 to S4

Tables: S1 to S11

References

**Supplementary methods**

*Stable isotope composition database*

Global Meteoric Water Line (GMWL) data were obtained from the Global Network of Isotopes in Precipitation (GNIP) database[1–4]. Local Meteoric Water Line (LMWL) data were taken from nearby stations (dating from 2005 ~ 2007) of the Chinese Network of Isotopes in Precipitation (CHNIP) database of the Institute of Geographic Sciences and Natural Resources Research (CAS) in Beijing. The Local Evaporation Line (LEL) was calculated according to previous studies[5, 6].

*CLSM image acquisition*

Syto 9-stained cells were imaged with an excitation wavelength of 488 nm and an emission wavelength of 525 nm. Cells stained with the PI were visualized at the excitation wavelength of 561 nm and emission wavelength of 595 nm.

*16S rRNA gene amplicon sequencing and analysis*

DNA sequencing was performed on an Illumina NovaSeq X Plus platform with an S Prime 100-cycle flow cell (2×100 base pairs). PCR amplification using the NEBNext Ultra II Q5 Master Mix, using the following program: 98 °C for 30 s; (98 °C 10 s, 53 °C 20 s, 72 °C 30 s) × 25; 72 °C for 2 min; hold at 12 °C. The Qsep400 system was used to evaluate library fragment size and quality, while the concentration was measured with the Qubit 4.0 fluorometer to ensure library quality. Libraries with a minimum total DNA input of 6.25 ng and an average fragment size of 350 bp were selected for sequencing. Negative controls were included at each step of the DNA workflow, including DNA extraction, PCR amplification, and library preparation, and no detectable amplification was observed in any of the negative controls.

The raw 16S rRNA gene sequences were processed using QIIME 2 (v2020.11.0). Raw paired-end reads were first processed with fastp for adapter trimming, removal of low-quality bases, and filtering based on a minimum read length of 70 bp. Cleaned reads were then imported into DADA2, where quality profiles were inspected to determine trimming parameters. Reads were filtered using trimLeft = c(25, 25) and truncLen = c(270, 200), and a maximum expected error threshold of maxEE = c(2, 3) was applied to retain high-quality sequences. Quality-filtered reads were used to learn an error model, followed by dereplication and sample-wise denoising to infer true biological sequences while removing amplification noise. Forward and reverse reads were merged using their overlapping regions to construct the ASV table, and sequences outside the expected length range were removed. Chimeric sequences were identified and removed using the consensus method implemented in removeBimeraDenovo. Read retention was tracked across all processing steps.

*Metagenomic sequencing and analysis*

The trimmed reads were mapped to the assembled scaffolds to calculate the sequence coverage[9]. A phylogenetic tree of the MAGs with more than 75% completeness and less than 10% contamination retrieved from this study was created based on a conserved marker gene set identified by checkM (v1.1.2)[14]. MAGs files and mapping files were inputted to anvi’o v7[15]. Mean coverage for the inner quartiles (Q2 and Q3) for each MAG was estimated by summarizing the metagenome profiles. The relative coverage for each MAG was calculated by dividing its coverage to the total coverage of all MAGs. Coding gene sequences (CDS) and their translations were predicted by Prodigal (v2.6) with “meta” option[16]. To determine the replication index of MAGs, the sequencing coverage resulted from read mapping files was used to calculate the index of replication (iRep)[17]. Annotation of the amino acid sequences was performed using several databases: KofamKOALA[18], GhostKOALA[19], eggNOG v5.0[20], HydDB[21], dbCAN2 (v2.0.11)[22], TCDB[23] and MEROPS (release 12.4)[24]. Additionally, putative genes encoding RubisCO, McrA, AssA/BssA, Cld and Nod were retrieved from the metagenome through BLASP queries against comprehensive custom database. Key metabolic marker genes were manually verified using BLASTP through the NCBI web server tool against the *nr* database. To predict the organismal oxygen phenotype, namely aerobic or anaerobic, protein sequences of the MAGs with completeness ≥75% and contamination ≤10% were analyzed for oxygen-utilizing enzymes using the classifier program Oxyphen which is based on machine learning strategy[25]. The 117 MAGs collectively recruited an average of 89.3% of total metagenomic reads (73.7% ~ 96.2%). For a complementary read‑based profiling approach, genes were predicted from the assembled scaffolds using Prodigal (v2.6), and a non‑redundant gene set was constructed using mmseqs2 (v15.6f452) with the easy‑cluster function. The oxygen phenotype prediction was also manually verified by the experimental data of the closest strains in the literature when available. The predicted optimal growth temperatures (predOGT) were calculated using a machine learning algorithm Tome[26]. 16S rRNA profiling in the metagenome was conducted by mapping metagenomic short reads to the SILVA SSU reference database v138 to assign the nearest taxonomic units using phyloFlash v3.4[27].

*Microbial community diversity analysis*

An abundance matrix was extracted from the ASVs table, and samples were assigned to their corresponding experimental groups based on metadata. PCoA was performed using classical multidimensional scaling (cmdscale), and the proportion of variance explained by each axis, as well as sample coordinates, was obtained. The resulting R^2^ and *p*-values were reported to indicate effect size and statistical significance. PCoA ordinations were visualized with 95% confidence ellipses using stat_ellipse.

*Redundancy analysis (RDA) and Mantel test*

Both the ASV abundance matrix and environmental variable matrix were standardized using Z-score transformation to reduce the influence of differences in scale and magnitude. Environmental variables that were aliased due to collinearity in the RDA model were projected onto the ordination space using the envfit function, which also assessed the statistical significance of each variable. Mantel *r* and *P* values were visualized in a correlation heatmap, with significant associations highlighted using curved linking lines to illustrate key ASV-environment relationships.

*ESI FT-ICR MS analysis*

Instrument settings were as follows: capillary voltage: 3900 V; nebulizer: 2.0 bar; dry gas: 4.0 L/min; dry temperature: 200 ℃; end plate offset: –800 V. For calibration, the Suwannee River Fulvic Acid (SRFA, 2R101N, International Humic Substances Society). The instrument was initially calibrated with sodium formate and subsequently recalibrated using a known mass series from 100 µg/mL SRFA, which provided a sufficient abundance of CHO-containing compounds to ensure mass accuracy within 0.2 ppm across the measured range. Molecular formula was assigned using MassAnalyzer (v1.0) software[28, 29].

*Data acquisition of UHPLC-MS/MS*

Data were acquired in data-dependent acquisition (DDA) mode, comprising 1 full scan followed by 10 MS/MS scans. Specifically, full scan MS spectra (67~1,000 amu) were recorded at a resolution of 70,000 (automatic gain control (AGC) target: 1×10^6^, maximum injection time: 100 ms). For data-dependent MS/MS (dd-MS/MS), a resolution of 17,500 was used with AGC target of 5×10^5^ and maximum injection time of 50 ms.

**Figures and Tables**

**Fig. S1.** **viscosity of shale oil samples from HY1, HY4 and HY7.** Temperature-dependent viscosity of raw shale oil (A) and dehydrated oil (B) samples.

**Fig. S2. CT scan of shale oil samples from three oil reservoirs.** (A) Spatial distribution of water droplets (blue) in the oil sample. (B) The size distribution of water droplets in the shale oil samples from HY1, HY4, and HY7. Pairwise comparisons were tested with pairwise Wilcoxon test, ***: *p* < 0.001.

**Fig. S3. Richness and simpson indexes of microbial communities in water samples of different sources.** (A) Richness index of microbial communities in water samples from different oil reservoirs. (B) Simpson index of microbial communities in water samples from different oil reservoirs. Statistical significance evaluated by PERMANOVA, ns: *p* > 0.05.

**Fig. S4.** **Relative sequencing coverages of MAGs groups based on their predicted functional roles.** MAGs with multiple functions were repeatedly assigned to different groups. Each dot represents a MAG, and the boxes indicate the means and standard errors. No significant difference was observed among function roles using a non-parametric Kruskal-Wallis analysis.

**Table S1.**

**Characteristics of the oil sample**

| Well number | HY1 | HY4 | HY7 |
| --- | --- | --- | --- |
| Depth (m) | 3810 ~ 4057 | 3405 ~ 3606 | 4151 ~ 4278 |
| TDS (mg/L) | 29205.45 | 28932.52 | 9243.87 |
| Density at 15 °C (g/cm^3^) | 0.8567 | 0.8646 | 0.7952 |
| API gravity | 32.90 | 32.07 | 46.36 |

**Table S2.**

**FTICR-MS data of the oil sample, extracted water, and water droplets.**

Green: Formula detected in samples from HY1;

Yellow: Formula detected in samples from HY4;

Bule: Formula detected in samples from HY7.

**Table S3.**

**Water content of shale oil samples from three oil reservoirs.**

| Oil reservoir | Water content (%) | Number of water droplets (/ml) |
| --- | --- | --- |
| HY1 | 7.8 ± 0.1 | 4.04 × 10^6^ |
| HY4 | 7.1 ± 0.2 | 1.46 × 10^6^ |
| HY7 | 2.3 ± 0.1 | 0.23 × 10^6^ |

**Table S4.**

**The size distribution of water droplets in shale oil samples.**

Data were obtained by the CT scan and further analyzed by AVIZO software.

**Table S5.**

**Ion concentrations of the gravitationally separated water (GSW), separated water (SW) and fracturing fluid from three shale oil reservoirs.**

| Component | GSW | | | SW | | | Fracturing fluid | |
| --- | --- | --- | --- | --- | --- | --- | --- | --- |
| (mg/L) | HY1 | HY4 | HY7 | HY1 | HY4 | HY7 | HY1&HY4 | HY7 |
| Formic acid | <0.07 | <0.07 | <0.07 | 13.3 | 1.53 | 8.84 | 3.03 | 1.28 |
| Acetic acid | 31 | 177 | 13 | 52.8 | 133 | 66 | 4.57 | 4.25 |
| Propionic acid | <0.17 | 12.1 | <0.17 | 0.35 | 2.8 | 11.2 | <0.17 | <0.17 |
| Butyric acid | <0.26 | <0.26 | <0.26 | <0.93 | <0.93 | 0.86 | <0.26 | <0.26 |
| Cl^-^ | 17013 | 16388 | 4457 | 17552 | 16602 | 5982 | 650 | 156 |
| NO_3_^-^ | 1.89 | 0.85 | 3.1 | 3.74 | 2.53 | 8.49 | 182 | 9.7 |
| NO_2_^-^ | 0.16 | 0.17 | 0.07 | 2.54 | 3.69 | <0.4 | 0.08 | 0.17 |
| CO_3_^2-^ | 11.3 | 12.6 | 29.5 | <0.10 | <0.10 | 207 | 19.1 | 22.6 |
| SO_4_^2-^ | 464 | 569 | 105 | 736 | 606 | 399 | 44.6 | 40.8 |
| S^2-^ | <0.08 | <0.08 | <0.08 | <0.26 | <0.26 | <0.5 | <0.08 | <0.08 |
| Na^+^ | 11004 | 11107 | 4283 | 10310 | 10419 | 5037 | 81.1 | 177 |
| NH_4_^+^ | 198 | 184 | 100 | 848 | 357 | <0.4 | 3.97 | 4.45 |
| K^+^ | 356 | 289 | 181 | 319 | 256 | 252 | 19.1 | 72.4 |
| Mg^2+^ | 12.1 | 30.8 | 11.2 | 12.9 | 34.1 | <0.2 | 3.02 | 1.31 |
| Ca^2+^ | 114 | 162 | 61 | 66.3 | 83.9 | <0.2 | 7.19 | 2.16 |

**Table S6.**

**Shannon index of microbial communities in isolated water droplets (WD) and separated water (SW) from different oil reservoirs.**

**Table S7.**

**Genome information of the MAGs (>50% complete, <10% contamination) used in this study**.

Taxonomy for all genomes is provided using GTDB-tk v1.3.0. The completeness and contamination of MAGs were estimated according to the number of single-copy marker genes using CheckM (v1.1.2). HQ MAGs are defined as those with completeness >90% and contamination <5%, which are also shaded in this table.

**Table S8.**

**Results of redundancy analysis (RDA) showing the scores, contributions, and significance of environmental variables on the first two constrained axes (RDA1 and RDA2).**

For each ASV, positive and negative associations with physicochemical variables are reported together with correlation coefficients and *p*-values. ASVs with taxonomic assignments and relative abundances across samples were also listed.

**Table S9.**

**Annotation of genes associated with specific metabolisms in 117 MAGs.**

The KO numbers of genes were assigned using a combined method of GhostKOLA, KofamKOALA and eggNOG-mapper V2.

**Table S10.**

**BLAST‑based identification of potential PflD (pyruvate formate‑lyase 2) homologs in metagenomic assemblies.**

The protein sequence of PflD from Archaeoglobus fulgidus DSM 4304 (GenBank accession: AAB89800.1) was used as the query against a database of predicted proteins from all assembled scaffolds (E‑value threshold = 10^-5^). Detected sequences represent candidate genes potentially involved in anaerobic hydrocarbon degradation pathways. HQ MAGs are shaded in gray.

**Table S11.**

**Differential KEGG Orthologs identified across HY1, HY4, and HY7 samples.**

Differential gene expression analysis was conducted using the DESeq2 package based on a negative binomial generalized linear model. The approach estimated and shrunk gene-specific dispersion via empirical Bayes methods, and significant changes were identified using the Wald test with Benjamini-Hochberg correction. Genes with an adjusted *p*-value<0.05 and |log2FC|>5 were considered significantly differentially expressed.

**Table S12.**

**UPLC-MS/MS data of water droplets from HY1, HY4 and HY7.**

The raw data were transformed into Progenesis QI (Waters, United States) for data analysis. Compounds were annotated. The classification of compounds was conducted using formulas to search against the Human Metabolome Database (HMDB).

**References**

1.	Gibson JJ, Birks SJ, Edwards TWD. Global prediction of δA and δ2H- δ18O evaporation slopes for lakes and soil water accounting for seasonality. *Global Biogeochem Cycles* 2008;22. https://doi.org/10.1029/2007GB002997

2.	Vystavna Y et al. Stable isotopes in global lakes integrate catchment and climatic controls on evaporation. *Nat Commun* 2021;12. https://doi.org/10.1038/s41467-021-27569-x

3.	Rozanski K, Araguás-Araguás L, Gonfiantini R. Isotopic Patterns in Modern Global Precipitation. 2013;1–36. https://doi.org/10.1029/GM078p0001

4.	A L, E S. Isotopic composition of precipitation in Italy: a first overall map. *J Hydrol (Amst)* 2003;270:75–88. https://doi.org/10.1016/S0022-1694(02)00281-0

5.	Zhang B et al. The interaction between surface water and groundwater and its effect on water quality in the Second Songhua River basin, northeast China. *Journal of Earth System Science* 2016;125:1495–1507. https://doi.org/10.1007/s12040-016-0742-6

6.	Zhang B et al. A study of the interrelation between surface water and groundwater using isotopes and chlorofluorocarbons in Sanjiang plain, Northeast China. *Environ Earth Sci* 2014;72:3901–3913. https://doi.org/10.1007/s12665-014-3279-5

7.	Quast C et al. The SILVA ribosomal RNA gene database project: Improved data processing and web-based tools. *Nucleic Acids Res* 2013;41:590–596. https://doi.org/10.1093/nar/gks1219

8.	Nurk S et al. MetaSPAdes: A new versatile metagenomic assembler. *Genome Res* 2017;27:824–834. https://doi.org/10.1101/gr.213959.116

9.	Langmead B, Salzberg SL. Fast gapped-read alignment with Bowtie 2. *Nat Methods* 2012;9:357–359. https://doi.org/10.1038/nmeth.1923

10.	Wu Y-W, Simmons BA, Singer SW. MaxBin 2.0: an automated binning algorithm to recover genomes from multiple metagenomic datasets. *Bioinformatics* 2016;32:605–607. https://doi.org/10.1093/bioinformatics/btv638

11.	Kang DD et al. MetaBAT 2: an adaptive binning algorithm for robust and efficient genome reconstruction from metagenome assemblies. *PeerJ* 2019;7. https://doi.org/10.7717/peerj.7359

12.	Alneberg J et al. Binning metagenomic contigs by coverage and composition. *Nat Methods* 2014;11:1144–1146. https://doi.org/10.1038/nmeth.3103

13.	Sieber CMK et al. Recovery of genomes from metagenomes via a dereplication, aggregation and scoring strategy. *Nat Microbiol* 2018;3:1–8. https://doi.org/10.1038/s41564-018-0171-1

14.	Parks DH et al. CheckM : assessing the quality of microbial genomes recovered from isolates , single cells , and metagenomes. *Genome Res* 2015;25:1043–1055. https://doi.org/10.1101/gr.186072.114.Freely

15.	Eren AM et al. Anvi’o: an advanced analysis and visualization platform for ‘omics data. *PeerJ* 2015;3. https://doi.org/10.7717/peerj.1319

16.	Hyatt D et al. Prodigal: prokaryotic gene recognition and translation initiation site identification. *BMC Bioinformatics* 2010;11:119. https://doi.org/10.1186/1471-2105-11-119

17.	Brown CT et al. Measurement of bacterial replication rates in microbial communities. *Nat Biotechnol* 2016;34:1256–1263. https://doi.org/10.1038/nbt.3704

18.	Aramaki T et al. KofamKOALA: KEGG Ortholog assignment based on profile HMM and adaptive score threshold. *Bioinformatics* 2020;36:2251–2252. https://doi.org/10.1093/bioinformatics/btz859

19.	Kanehisa M, Sato Y, Morishima K. BlastKOALA and GhostKOALA: KEGG Tools for Functional Characterization of Genome and Metagenome Sequences. *J Mol Biol* 2016;428:726–731. https://doi.org/10.1016/j.jmb.2015.11.006

20.	Cantalapiedra CP et al. eggNOG-mapper v2: Functional Annotation, Orthology Assignments, and Domain Prediction at the Metagenomic Scale. *Mol Biol Evol* 2021;38:5825–5829. https://doi.org/10.1093/molbev/msab293

21.	Sondergaard D, Pedersen CNS, Greening C. HydDB: A web tool for hydrogenase classification and analysis. *Sci Rep* 2016;6:1–8. https://doi.org/10.1101/061994

22.	Zhang H et al. DbCAN2: A meta server for automated carbohydrate-active enzyme annotation. *Nucleic Acids Res* 2018;46:W95–W101. https://doi.org/10.1093/nar/gky418

23.	Saier MH et al. The transporter classification database (TCDB): 2021 update. *Nucleic Acids Res* 2021;49:D461–D467. https://doi.org/10.1093/nar/gkaa1004

24.	Rawlings ND et al. The MEROPS database of proteolytic enzymes, their substrates and inhibitors in 2017 and a comparison with peptidases in the PANTHER database. *Nucleic Acids Res* 2018;46:D624–D632. https://doi.org/10.1093/nar/gkx1134

25.	Jabłońska J, Tawfik DS. The number and type of oxygen-utilizing enzymes indicates aerobic vs. anaerobic phenotype. *Free Radic Biol Med* 2019;140:84–92. https://doi.org/10.1016/j.freeradbiomed.2019.03.031

26.	Abdulkarim MA, Muxworthy AR, Fraser A. High temperature susceptibility measurements: A potential tool for the identification of oil-water transition zone in petroleum reservoirs. *Front Earth Sci (Lausanne)* 2022;10. https://doi.org/10.3389/feart.2022.973385

27.	Gruber-Vodicka HR, Seah BKB, Pruesse E. phyloFlash: Rapid Small-Subunit rRNA Profiling and Targeted Assembly from Metagenomes. *mSystems* 2020;5. https://doi.org/10.1128/msystems.00920-20

28.	Zhang Z. Large-scale identification and quantification of covalent modifications in therapeutic proteins. *Anal Chem* 2009;81:8354–8364. https://doi.org/10.1021/ac901193n

29.	Zhang Z, Zhang A, Xiao G. Improved protein hydrogen/deuterium exchange mass spectrometry platform with fully automated data processing. *Anal Chem* 2012;84:4942–4949. https://doi.org/10.1021/ac300535r
